# Tomato Mutants Reveal Root and Shoot Strigolactone Involvement in Branching and Broomrape Resistance

**DOI:** 10.3390/plants13111554

**Published:** 2024-06-04

**Authors:** Uri Karniel, Amit Koch, Nurit Bar Nun, Dani Zamir, Joseph Hirschberg

**Affiliations:** 1 Department of Genetics, Alexander Silberman Institute of Life Sciences, The Hebrew University of Jerusalem, Jerusalem 9190401, Israel; urikarniel@gmail.com (U.K.);; 2 Robert H. Smith Institute of Plant Sciences and Genetics, The Hebrew University of Jerusalem, Rehovot 7610001, Israel; amitkoch3@gmail.com (A.K.); dani.zamir@mail.huji.ac.il (D.Z.)

**Keywords:** strigolactone, tomato, mutations, *Phelipanche aegyptiaca*, parasitic plants

## Abstract

The phytohormones strigolactones (SLs) control root and shoot branching and are exuded from roots into the rhizosphere to stimulate interaction with mycorrhizal fungi. The exuded SLs serve as signaling molecules for the germination of parasitic plants. The broomrape *Phelipanche aegyptiaca* is a widespread noxious weed in various crop plants, including tomato (*Solanum lycopersicum*). We have isolated three mutants that impair SL functioning in the tomato variety M82: *SHOOT BRANCHING 1* (*sb1*) and *SHOOT BRANCHING 2* (*sb2*), which abolish SL biosynthesis, and *SHOOT BRANCHING 3* (*sb3*), which impairs SL perception. The over-branching phenotype of the *sb* mutants resulted in a severe yield loss. The isogenic property of the mutations in a determinate growth variety enabled the quantitative evaluation of the contribution of SL to yield under field conditions. As expected, the mutants *sb1* and *sb2* were completely resistant to infection by *P. aegyptiaca* due to the lack of SL in the roots. In contrast, *sb3* was more susceptible to *P*. *aegyptiaca* than the wild-type M82. The SL concentration in roots of the *sb3* was two-fold higher than in the wild type due to the upregulation of the transcription of SL biosynthesis genes. This phenomenon suggests that the steady-state level of root SLs is regulated by a feedback mechanism that involves the SL signaling pathway. Surprisingly, grafting wild-type varieties on *sb1* and *sb2* rootstocks eliminated the branching phenotype and yield loss, indicating that SL synthesized in the shoots is sufficient to control shoot branching. Moreover, commercial tomato varieties grafted on *sb1* were protected from *P*. *aegyptiaca* infection without significant yield loss, offering a practical solution to the broomrape crisis.

## 1. Introduction

Strigolactones (SLs) are a group of conserved carotenoid-derived hormones present across all land plants. They were first characterized as potent stimulant crystalline compounds that induced the germination of the parasitic weed *Striga lutea* [1]. Further research showed that SLs are involved in plant development and responses to biotic and abiotic stresses and rhizosphere signaling [2,3,4,5]. One of the most important roles of SLs is to suppress axillary bud growth and shoot branching [6,7,8,9]. SLs also affect root architecture [10,11] and are involved in other processes, ranging from seed germination to senescence [4,12,13,14]. Recent evidence has revealed crosstalk mechanisms between SLs and other phytohormones [15,16].

Below the ground, SLs are exuded from roots, stimulating various processes in the rhizosphere. An important role of SLs is the establishment of a beneficial symbiosis with arbuscular mycorrhizal fungi through promoting root colonization and hyphal branching, which improves the plant’s mineral nutrition [5]. It was shown that a deficiency in the primary nutrients phosphate and nitrogen induces SL biosynthesis and response [17,18]. SLs promote plant defense against root-knot nematodes in tomato (*Solanum lycopersicum*) by influencing the accumulation of the phytohormones jasmonic acid and abscisic acid in the roots [19].

SLs exuded by the roots stimulate the germination of several parasitic plant species, most of which belong to the *Orobanchaceae* (broomrapes) family. This family comprises the Striga, Orobanche, and Phelipanche genera, among the most numerous holoparasitic weeds responsible for severe damage to crop yield worldwide [20]. *Phelipanche aegyptiaca* spp. (Egyptian broomrape) is a widespread noxious weed in tomato fields in Africa, the Middle East, and the Mediterranean [21]. This parasitic plant can cause severe yield loss ranging from 5% to 100% [22]. The life cycle of *P. aegyptiaca* starts with seed germination in response to SL released by the host plant, followed by attachment to the host roots and haustorium formation toward the vascular system of host roots, resulting in a compatible interaction. The inflorescence of the parasitic plant emerges from the soil and develops flowers that produce a massive number of seeds [23].

**Figure 1:**
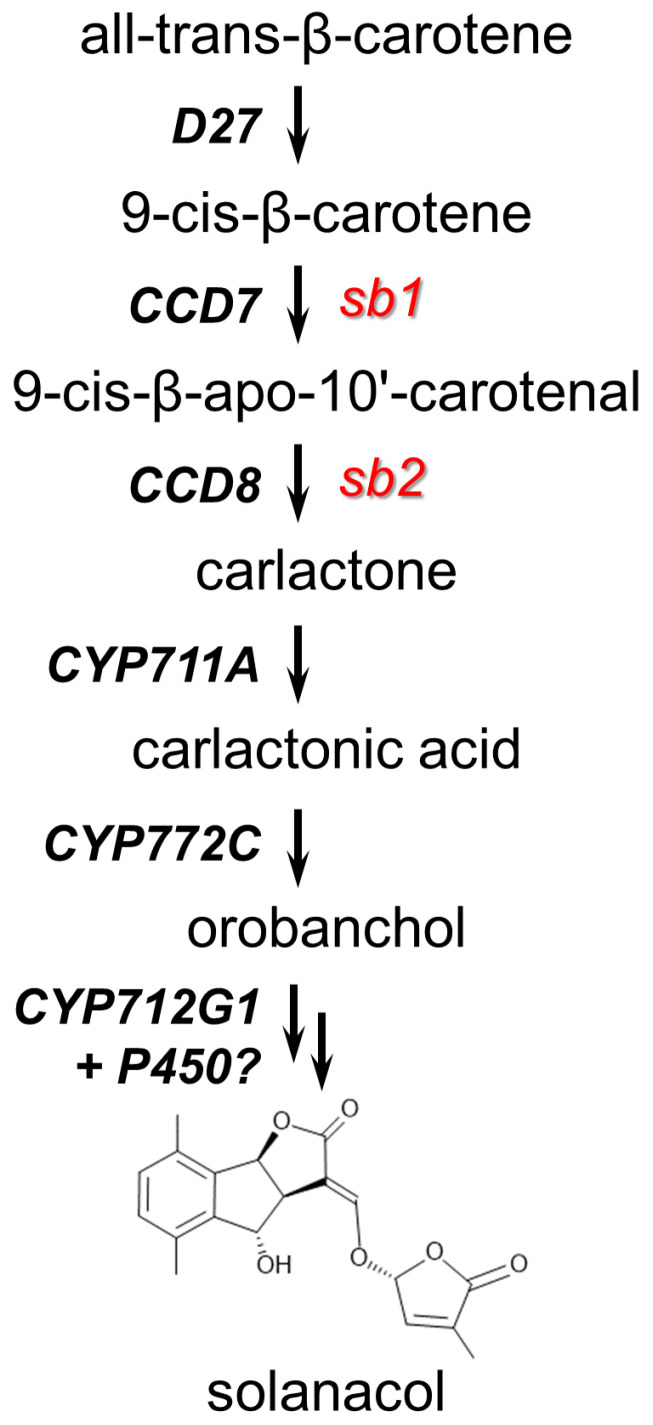
The strigolactone biosynthesis pathway in tomato. D27—β-carotene isomerase; CCD7—carotenoid cleavage dioxygenase 7; CCD8—carotenoid cleavage dioxygenase 8; CYP711A (MAX1), CYP722C, and CYP712G1 are P450 oxygenases; *sb1* and *sb2* are mutations of the genes *SlCcd7* and *SlCcd8*, respectively.

The initial steps in SL biosynthesis in plants occur in the plastids. This process starts with the isomerization of the C9-C10 double-bond of all-*trans* β-carotene to produce 9-*cis*-β-carotene, catalyzed by the carotene isomerase DWARF27 (D27) [24]. Further reactions, catalyzed by carotenoid cleavage dioxygenase 7 (CCD7) and CCD8, convert 9-*cis* β-carotene to carlactone, the common precursor for all the divergent active molecules in the SL family (Alder et al. 2012) [25,26]. Subsequent reactions in the cytosol are catalyzed by cytochrome P450 enzymes, which convert carlactone into carlactonic acid and functional SLs through hydroxylation and oxidation reactions [24,27,28,29,30] (Figure 1).

The perception pathway of SLs starts with their binding by the receptor DWARF14 (D14) [31,32]. This binding leads to the recruitment of the F-box protein MORE AXILLARY BRANCHES 2 (MAX2), which targets the repressor proteins DWARF53 and SUPPRESSOR OF MAX2 1-LIKE (SMXL) for ubiquitination and subsequent degradation in the proteasome, resulting in the activation of various SLs’ downstream target genes [33]. The receptor D14, which belongs to the α/β-hydrolase enzyme superfamily, is conserved in all land plants [13,34] and has recently been characterized in tomato (*Solanum lycopersicum*) [35].

Tomato is a major horticultural crop of global importance, and the parasitic weed *P. aegyptiaca* endangers its cultivation in vast areas worldwide [20]. Several strategies have been developed to cope with broomrapes [21]. Most use chemicals, including herbicides, soil fumigation that kill the parasites, and field treatment before cultivation with SL analogs, in a process known as ‘suicidal germination’ [36,37]. Apart from these methods, breeding *P. aegyptiaca*-resistant varieties, based on the host’s low exudation of the SL stimulants, has been attempted [22,38,39,40,41,42,43,44]. Some were based on impairing the functions of the carotenoid cleavage enzymes CCD7 and CCD8, which are involved in the SLs’ biosynthesis pathway.

Here, we describe identifying and characterizing isogenic tomato mutants impaired in the CCD7 and CCD8 enzymes and a mutant in the SL-receptor D14. The molecular and physiological characterization of these mutants shed new light on SLs functioning in tomato, the regulation of their synthesis, and their effects on broomrape resistance.

## 2. Results

### 2.1. Isolation and Molecular Characterization of SL Mutants

**Figure 2:**
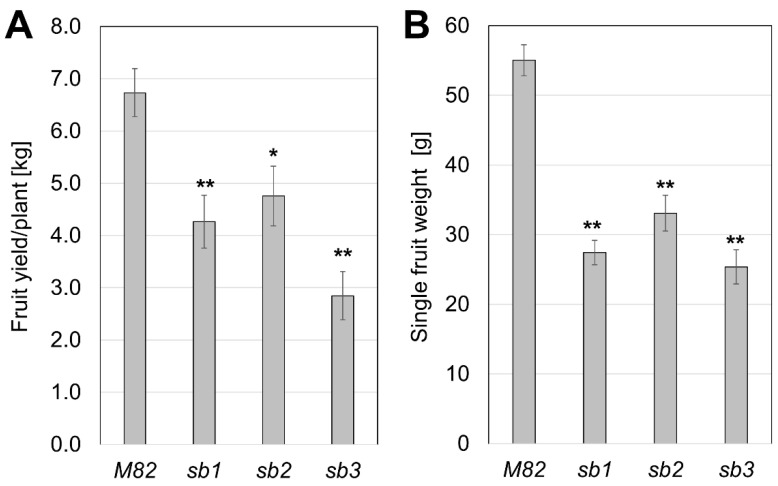
Yield parameters in field-grown SL mutants. Fruit yield [kg/plant] (**A**) and single fruit weight [g] (**B**) at the time of harvest of the wild type (M82) and the isogenic mutants *sb1*, *sb2*, and *sb3* (*n* ≥ 10, ±SE, * *p* < 0.05, ** *p* < 0.01).

The collection of ethylmethane sulfonate (EMS)-mutagenized tomato plants (*Solanum lycopersicum* cv M82) [45] was screened for mutants with alternative growth habits. Three mutants with increased shoot branching were identified (Figure S1A) and named *SHOOT-BRANCHING1* (*sb1*), *SHOOT-BRANCHING2* (*sb2*), and *SHOOT-BRANCHING3* (*sb3*). The distinctive phenotype suggested that these mutants were impaired in strigolactone (SL) functions. The over-branching phenotype caused a significant reduction in total fruit yield due to the decrease in fruit size and fruit set (Figure 2 and Figure S1B).

**Figure 3:**
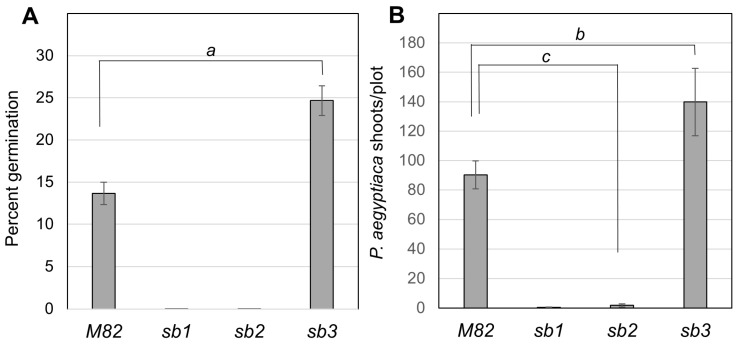
Root strigolactone and broomrape susceptibility of SL mutants. (**A**) The quantification of SL in roots of M82 and the mutants *sb1*, *sb2,* and *sb3* based on the germination rate of *P. aegyptiaca* seeds induced by root extracts. Germination was recorded after 14 days. There was no germination in *sb1* and *sb2* assays. Data represent an average of three independent replications (*n* = 3, ±SE, *a*, *p* < 0.05). (**B**) Infection of field-grown tomato mutants *sb1*, *sb2* and *sb3* and the wild-type M82 by *P. aegyptiaca* (*n* = 5, ±SE, *b*, *p* = 0.096; *c*, *p* < 0.001).

The amount of SL in the mutants’ roots was estimated using a bioassay of the germination of *P. aegyptiaca* seeds (Materials and Methods). The results showed that root extracts from *sb1* and *sb2* plants starved for phosphate did not induce the germination of *P. aegyptiaca* compared with the wild-type line M82 (Figure 3A). In contrast, the root extract from *sb3* increased the germination rate of *P. aegyptiaca* by two-fold compared to M82 (Figure 3A). These results explain the high susceptibility of the *sb3* plant to infection by *P. aegyptiaca* in the field (Figure S2). The response of the *sb* mutants to infection by *P. aegyptiaca* was measured in a field infested with seeds of this parasite by the number of broomrape inflorescences per plot. No broomrape inflorescences were found in mutants *sb1* and *sb2*, indicating that they were resistant to infection by *P. aegyptiaca* (Figure 3B). These results agree with the finding that *sb1* and *sb2* lack SLs. In contrast, the infection of *sb3* plants was more than 50 percent higher than that of wild-type M82 plants. The increased susceptibility of *sb3* plants to *P. aegyptiaca* corresponds to the higher level of SL in this mutant’s roots (Figure 3A,B).

Mutations in tomato that impair strigolactone biosynthesis have previously been reported in the carotenoid cleavage dioxygenase enzymes *Sl*CCD7 (Solyc01g090660.2) and SlCCD8 (Solyc08g066650.2) [7,40,44,46,47,48]. The genes *SlCcd7* and *SlCcd8* were sequenced in the *SHOOT-BRANCHING* mutants and compared with M82. The sequence data showed that the mutations in *sb1* and *sb2* are in *SlCcd7* and *SlCcd8*, respectively (Table 1). The gene *SlCcd7* encodes a polypeptide with 663 amino acid residues and a molecular weight of 75 kDa. The gene *SlCcd7* from the mutant *sb1* contains two mutations that alter the splice site of exon #7 (Figure S3A). An alternative splicing event in intron #6 creates a seven-nucleotide deletion in the mRNA, leading to a frameshift mutation and a truncated polypeptide (Figure S3B). CCD8 in tomato is a 64.7 kDa polypeptide with 579 amino acid residues. A point mutation of G to A in position 2659 of *SlCcd8* from *sb2* creates a missense mutation that changes glutamate to lysine in mutant *sb2* (Figure S4A,B).

In contrast to *sb1* and *sb2*, the third mutant *sb3* exhibited high shoot branching and a higher SL concentration. This finding suggested that *sb3* is involved not in the SLs’ biosynthesis but in one of the genes that participated in the SLs’ signaling pathway. Three major components are involved in the SLs’ perception pathway: the a/b-fold hydrolase D14, the F-box protein MAX2, and the repressor protein D53 [25,49]. The recessive nature of *sb3* eliminated the possibility of gain of function mutation in this mutant’s D53 repressor. Furthermore, comparing the two tomato orthologous genes, Solyc07g055120 and Solyc12g010900, from *sb3* and M82 indicated no polymorphism in their sequences. However, a mutation in *sb3* was discovered in the gene *Sl*Dwarf14 (*sl*D14) (Solyc04g077860), which encodes a 29.8 kDa protein with 267 amino acid residues (Table 1, Figure S5A). The G to A mutation at position 2582 in the *sl*Dwarf14 gene from *sb3* eliminates the splice site in exon #2, resulting in 17 nucleotides being deleted in the mRNA caused by alternative splicing that produces a truncated protein (Figure S5B).

**Figure 4:**
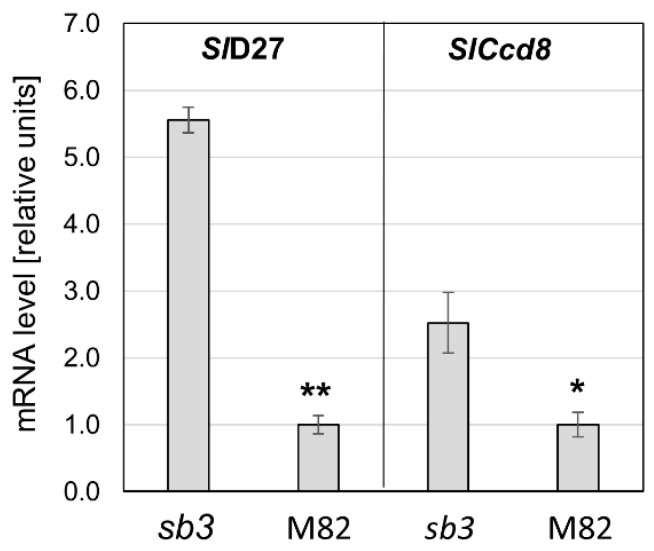
The quantification of the mRNA of the genes encoding slD27 and SlCCD8 in the roots of the mutant *sb3* and the wild-type M82. (*n* = 3, ±SE, * *p* < 0.05, ** *p* < 0.01).

The higher concentration of SL in *sb3* suggested that strigolactone signaling is involved in regulating the steady-state level of the phytohormone. Therefore, the expression of the genes *SlD27*, the first step in strigolactone biosynthesis, and *SlCcd8* were analyzed in the roots of *sb3* and WT (M82). Despite the SLs’ abundance, the expression of these genes was higher in *sb3* compared with M82 (Figure 4). Since the mutation in *sb3* eliminates the SL receptor D14, this result suggests a feedback regulation of the SL biosynthesis genes operating through SL perception and signal transduction.

### 2.2. The Phenotype of sb Mutants in Grafting Experiments

**Figure 5:**
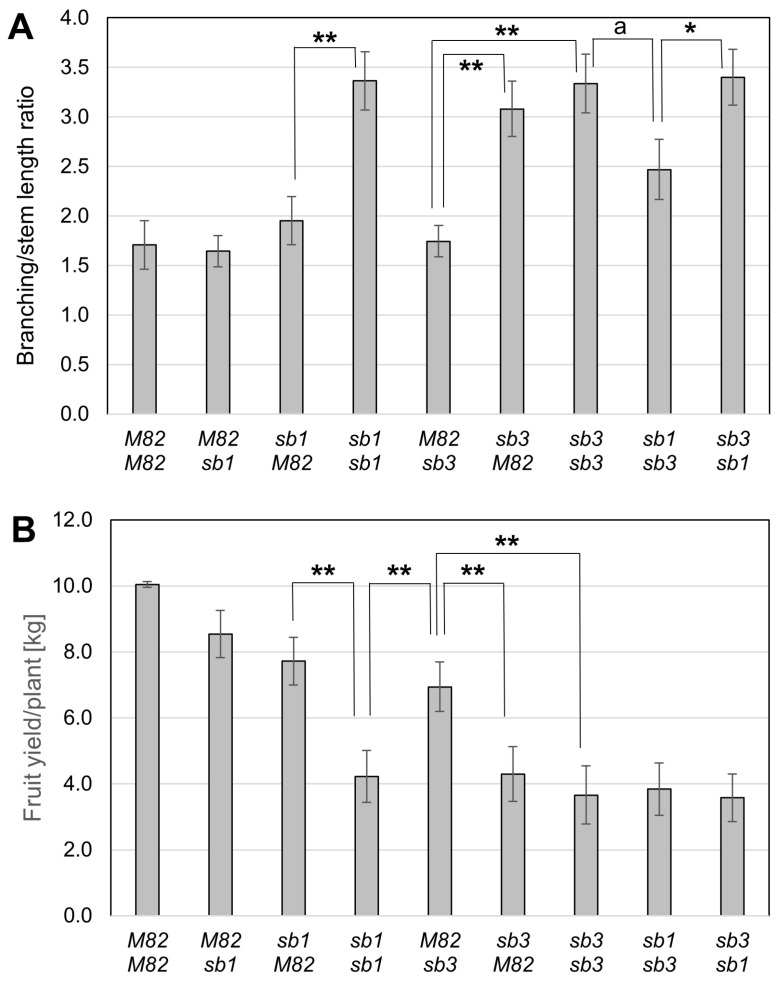
Root and shoot contribution to branching (**A**) and fruit yield (**B**) in plants with different grafting combinations (scion/rootstock) of the mutants *sb1* and *sb3* and the wild-type M82. The branching rate was determined as the number of branches per shoot length in centimeters. At least three plants from each grafting combination were characterized for branching and ten for yield (±SE, a = 0.0505, * *p* < 0.05, ** *p* < 0.01).

Different grafting combinations using the *sb* mutants were carried out to estimate the contributions of roots and shoots to the SL in the plant. In these experiments, the SL mutants *sb1* and *sb3* and the wild type (M82) served as rootstocks and scions in all combinations, and several morphologic traits were measured (Figure S6). The parameter with the highest correlation to the branching phenotype was the ratio between the number of branches and the stem length (Figure 5A and Table S1). As expected, the grafting of *sb1* scion on *sb1* rootstock exhibited the typical over-branching phenotype. However, reciprocal grafting between the wild-type M82 and the *sb1* mutant did not significantly impact branching, suggesting that the shoot compensated for the SL deficiency in the roots, and vice versa (Figure 5A). The over-branching phenotype due to the inhibition of SL perception seen in the self-grafted *sb3* mutant was restored when M82 was grafted on *sb3* rootstock but not in the reciprocal grafting. This result indicates that the SL regulation on the branching is confined to the shoot SL signal transduction, lacking in the *sb3* scion. The yield parameters of the grafted plants generally corresponded to the degree of branching despite minor deviations that could reflect other unknown effects of the SLs on fruit or root development.

**Figure 6:**
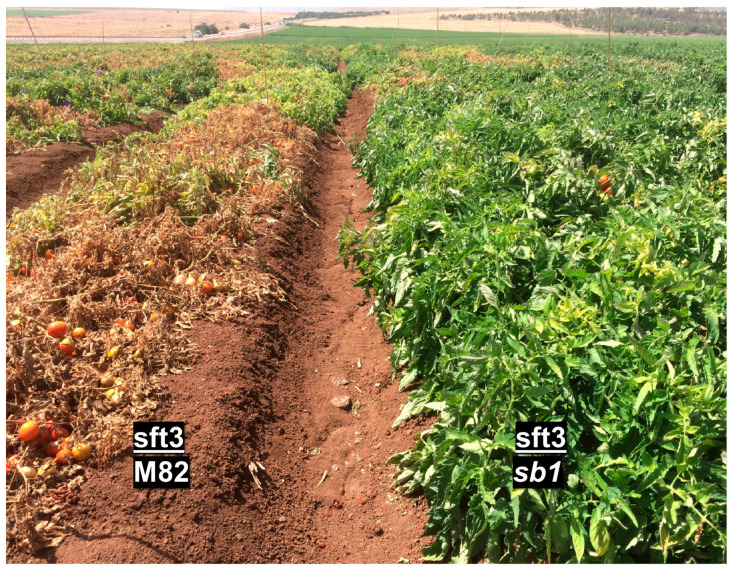
The resistance to *P. aegyptiaca* of grafted tomato plants. The cultivated tomato variety sft3 was grafted on *sb1* (**right**) or M82 (**left**) rootstocks. Plants were grown in a highly infested field in Ein Harod. The picture was taken two weeks before harvest.

**Figure 7:**
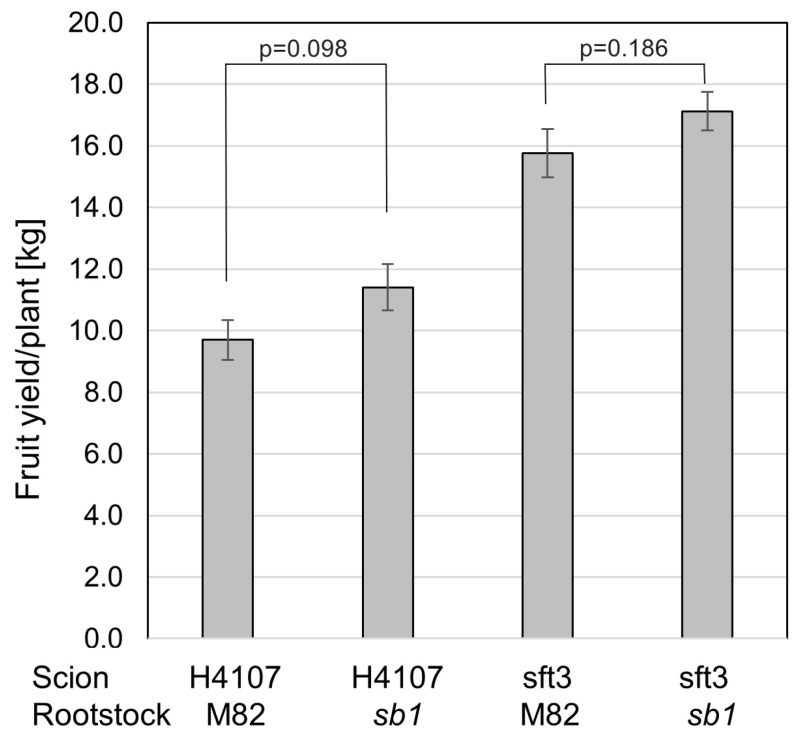
The performance of *sb1* as a rootstock compared with M82 in commercial tomato varieties in a field not infested by *P. aegyptiaca*. The total fruit yield of field-grown commercial varieties sft3 and H4107 grafted on *sb1* or M82 rootstock. (*n* = 18, ±SE).

We have observed that plants of mutants *sb1* and *sb2* grown in the *P. aegyptiaca*-infested field were not infected. This phenomenon can be attributed to the lack of SLs in these mutants. Therefore, we tested the resistance to *P. aegyptiaca* of a wild-type tomato variety grafted on *sb1* rootstock in a *P. aegyptiaca*-infested field. As illustrated in Figure 6, *sb1* rootstock rendered resistance to *P. aegyptiaca* in an infected field where wild-type plants are infected and eventually die. To evaluate the effectiveness of *sb1* as a rootstock for commercial tomato varieties, scions from the commercial varieties sft3 and H4107 were grafted onto *sb1* rootstock, and their yields were tested under standard horticulture conditions in a non-infested field (Figure 7). The fruit yield of plants grafted onto *sb1* was not significantly different from that of plants grafted onto M82. Therefore, we conclude that the mutant rootstock caused no yield loss.

## 3. Discussion

### 3.1. Isogenic Mutations in Strigolactone Synthesis and Perception

Strigolactones are a class of plant hormones that regulate various aspects of plant growth and development, including branching and root architecture, and play crucial roles in interactions between soil organisms and roots. In the present study, we identified and characterized loss-of-function mutations in tomato (*S. lycopersicum* cv. M82) that impair strigolactone biosynthesis, *sb1* and *sb2*, and perception, *sb3*. The mutations *sb1* and *sb3* in the *SlCcd7* and *SlD14* genes occurred at splicing sites, leading to aberrant transcripts that create early stop codons. The mutation *sb2* in the *Sl*Ccd8 gene causes a substitution of the negatively charged amino acid glutamic acid with the positively charged lysine at position 529 of the CCD8 protein. This glutamic acid residue is conserved in all CCD8 proteins examined in monocots and dicots [50], so the substitution likely impairs the enzymatic activity. The original mutants obtained through EMS mutagenesis [45] were backcrossed with the parental M82 wild-type line, and the characteristic over-branching phenotype co-segregated with the mutations in the respective genes among all F2 offspring in a typical 3:1 ratio. Mutations in SL biosynthesis genes in tomato have previously been described [7,38,40,42,48,51,52]. However, these mutants were isolated in different lines with diverse genetic backgrounds, making phenotypic characterizations of quantitative effects impossible to compare. In this study, we analyzed three mutations that affect SL functioning in an entirely isogenic background, allowing for accurate comparisons of the specific impact of SL on growth and agronomic traits.

The over-branching in the SL mutants was accompanied by a reduction in total fruit yield, which was partly caused by a significant decrease in fruit size (Figure 2). It is unclear whether the reduction in fruit size is a direct effect of the lack of SL signaling on fruit development or a result of a different allocation of photosynthates between the fruits and the enlarged vegetative organs of the plant. The latter option is supported by the grafting experiments, where the fruit yield was inversely proportional to the degree of branching in all grafting combinations (Figure 5 and Table S1).

The mutation *sb3* in the SL receptor D14 exposed differences between the absence of SLs and impairment in SL perception and signaling. A lack of strigolactones in the mutants *sb1* and *sb2* reduced fruit yield by 33 percent, compared with a 45 percent reduction when the SL perception was eliminated in *sb3*. The more severe effect in the absence of the D14 receptor can be attributed to the distinct influence on gene expression when the D14 protein is present but not induced by SLs compared to the condition where it is absent. The most likely explanation relates to the fact that perception of strigolactones by D14 requires the interaction with the F-box protein MAX2 to target proteins, such as SMXL/D53 repressor of SL signaling, for ubiquitin-dependent degradation [4,28,32,53]. Although strigolactones facilitate the interaction of D14 with MAX2, a residual small interaction may occur in the absence of SL. Other explanations might be feasible if the D14 protein serves other yet unknown functions unrelated to SL or if the higher SL concentrations in *sb3* (Figure 3) cause detrimental effects through a D14-independent mechanism.

It was demonstrated that the D14 receptor protein degrades SL upon perception [32]. The elevated concentration of SL in the *sb3* roots, which was also manifested by a higher infestation of *P. aegyptiaca* in the field (Figure 3), can be attributed in part to the lack of SL hydrolysis by D14. However, as seen in Figure 4, the transcript levels of SL-biosynthesis genes Sl*D27* and Sl*Ccd8* were 5- and 2.5-fold higher, respectively, when D14 signaling was blocked. These data strongly suggest that increased synthesis of SL underlies the higher SL levels in *sb3* roots, suggesting a feedback mechanism controlling the steady-state level of SL that involves SL signaling and the regulation of gene expression. This conclusion is supported by related phenomena observed in other plant species. In a D14 mutant in rice (*Oryza sativa*), the levels of epi-5-deoxystrigol in the roots were higher than in the wild type [54]. Mutations in the genes for D53, a repressor of SL signaling, led to the upregulation of D10, an orthologue gene of CCD8 [55]. In the *rms2* (D53 orthologue) mutant in pea (*Pisum sativum*), the expression of RMS5 (CCD7 orthologue) and RMS1 (CCD8 orthologue) genes was elevated [56]. In Arabidopsis, mutations in MAX2 (D53 orthologue) and D14 enhanced the expression of the SL biosynthesis genes MAX3 (CCD7 orthologue) and MAX4 (CCD8 orthologue) [28,57]. It was shown in rice that a paralog of the SL receptor D14, *WARF14-LIKE* (*D14L*), positively regulates SL biosynthesis [58].

### 3.2. Graft Transmissible Effects of SL

The over-branching phenotype of the SL-deficient mutant *sb1* mostly disappeared when it was grafted onto a wild-type rootstock (Figure 5A, Table S1), supporting the notion that the roots are the primary site of SL biosynthesis, which are transported acropetally to the shoots through the xylem [28,47,59]. Similar results were previously reported in other plant species [28,59,60,61,62,63]. Over-branching, accompanied by loss of fruit yield, was also recovered in a wild-type (M82) scion grafted onto the SL-deficient rootstock of *sb1* (Figure 5 and Table S1). This result indicates that SLs constitutively synthesized in shoots at >100-fold lower levels than in the roots [59] can compensate for SL-deficient roots and comply with the finding that SL synthesized in shoots control axillary bud outgrowth in apple (Malus × domestica) [64].

In contrast, M82 rootstocks did not complement the branching phenotype of *sb3* (Figure 5), indicating that lack of SL signaling in the shoot determines the over-branching phenotype regardless of SL levels. Grafting experiments in peas showed that the shoot branching phenotype of a scion lacking D14 was partially rescued by WT rootstocks [65,66]. It was demonstrated that the D14 protein in pea is transmissible from roots to shoots [66]. Our data indicate that, similar to petunia and Arabidopsis [62,67], this phenomenon is not observed in tomato.

The branching index of the grafting combination *sb1*/*sb3* was lower than in each mutation individually despite the higher SL concentration in *sb3* roots (Figure 5). However, the fruit yield loss was the same (Figure 5 and Table S1). A possible explanation for this inconsistency is that the higher SL concentration in the *sb3* roots also exists in the *sb1* lower parts of the grafted shoots, where the wild-type D14 receptor properly transduces it to limit branching. However, SLs involved in fruit development are solely provided by the shoots and are deficient in *sb1*.

### 3.3. A Genetic Solution to Broomrape Infestation

The Egyptian broomrape (*Phelipanche aegyptiaca*) is a highly damaging parasitic weed that attacks the roots of various crops, including tomato. *P. aegyptiaca* infestations severely damage tomato plants and lead to devastating yield losses [20]. Various methods have been used to cope with *P. aegyptiaca* infections in tomato fields [21,22], but these methods often come with economic and environmental costs associated with the use of chemicals. Developing tomato varieties resistant to *P. aegyptiaca* is a promising approach for improving tomato agriculture by reducing yield losses, minimizing environmental impacts, and promoting sustainable and efficient farming practices.

Strigolactones are critical to parasitic weed infestation. Broomrape seeds require the presence of strigolactones in the soil to trigger their germination and subsequent attachment to the host plant’s roots. Plant mutants lacking SL or containing altered SL composition are less susceptible or even resistant to broomrape ([39,42,43,48,68,69,70] and Figure 3). Since SL in the soil is produced by the plant and exuded into the soil from the roots, SL-deficient roots can be used as rootstocks for plants of elite varieties. However, SL deficiency also causes severe yield loss due to the over-branching phenotype. The grafting experiments indicate that SL-deficient rootstock provides *P. aegyptiaca* resistance to wild-type scion (Figure 6), while the wild-type shoot compensates for the adverse influence on branching and fruit yield (Figure 5). The field trial of commercial elite tomato varieties grafted on *sb1* rootstock proves that lack of strigolactones in the roots does not affect the yield (Figure 7). This result indicates that any potential detrimental effects on root architecture in *sb1* rootstock were not manifested by loss of yield, and the lack of SL-dependent communication between roots and beneficial soil microbes for enhancing nutrient uptake is irrelevant in a well-irrigated and fertilized field. Given that these roots confer resistance to broomrape, this method offers an effective solution for growing tomatoes in fields infected with *P. aegyptiaca*.

## 4. Materials and Methods

### 4.1. Plant Material and Growth Conditions

Tomato cultivar M82 served as a reference ‘wild type’. Seeds from that variety were treated with ethylmethane sulfonate (EMS) or fast neutron bombardment [45]). Visual screening of M2 plants identified several mutants with over-branching phenotypes. Following the measurement of the resistance level against *P. aegyptiaca*, infection in an inoculated field was established using the identified over-branching lines. Three mutant lines, *sb1*, *sb2*, and *sb3*, were isolated and further studied in this research. The commercial hybrids sft3 and H4107 used as scions in this study were obtained from Prof. Dani Zamir’s laboratory. Root extracts for qPCR and *P. aegyptiaca* germination bioassay were performed from five-week-old hydroponically grown plants in Hoagland medium in the greenhouse. For the bioassay analysis, the Hoagland medium was changed to Hoagland without phosphate for four days, followed by a water-only medium for three days to elevate the SL synthesis in Pi-starved condition.

The field trials presented in this study were performed during three growing seasons. In 2016 and 2017, they were conducted in the Eden research station and the Gadash Ein Harod. In 2018, the field trials were conducted at the Western Galilee Experimental station in Akko and Eden research station. The Akko experiments were performed in a wide-spacing planting density of one plant per 1 m^2^. The plants in the Eden experiment were grown in plots of 27 plants per 5 m^2^. Seedlings were grown in the greenhouse for 35 days and then transplanted to the field at the beginning of March in Eden and April in Akko.

Grafting experiments were conducted during the summer of 2018 in the Western Galilee experimental station in Akko, Israel. Grafted plants were prepared by Hishtil Nursery (Ashkelon, Israel, URL: https://www.hishtil.com/ accessed on 3 June 2024), as described [46]. Twenty-one days post-germination, sterile-grown seedlings were sectioned within the hypocotyl region, and the combinations of scion and rootstock were aligned to form a graft union. The grafted plants were transplanted to the field in April. The experiment examined individual plants in a completely randomized design in two blocks, and plant experiments were represented by a minimum of 15 replicates in each block. M82, *sb1*, and *sb3* seedlings were used for reciprocal and self-grafting as a control. Two types of measurements were performed. The first, which included vegetative and growth characteristics, was performed about 40 days after planting. The second, which included yield and fruit traits, was performed during harvesting, about 100 days after planting.

### 4.2. DNA Extraction and Sequencing

DNA was extracted from young tomato leaves of approximately 15 mg, as previously described [71]. Genomic DNA of the genes *SlCcd7* (Solyc01g090660.2), *SlCcd8* (Solyc08g066650.2), and *slDWARF14* (Solyc04g077860.2) was amplified via PCR from total genomic DNA, using a Readymix kit (PCR-Ready^TM^ High Yield, Syntezza Biosciences, Jerusalem, Israel). These genes’ whole DNA lengths were sequenced and compared to the *Solanum lycopersicum* reference genome (build SL2.40 in URL: http://solgenomics.net/). The mutations that located in these genes were identified by sequencing using the following primers: 5′-AGTGTCTTTTAGCACCACATGT-3′ (Forward) and 5′-CTTCAAGTCTTGCAACTACTTCA-3′ (Reverse) for *SlCcd7*, 5′-CAGAACAGGGCCAAATGACC-3′ (Forward) and 5′-ACCAAGGTCAGCTTCTTTTCC-3′ for *SlCcd8*, and 5′-CCTTAGTTTATGTTTGACAAAATTCAT-3′ (Forward) and 5′-CAAACAATGTTATGTCTGGTCTCA-3′ (Reverse) for *slDWARF14*.

### 4.3. RNA Extraction, cDNA Sequencing, and Measurement of mRNA with Quantitative Real-Time RT-PCR

RNA extraction from hydroponically grown tomato roots was extracted from approximately 200 mg tissue with TRI Reagent RNA isolation reagent (Sigma-Aldrich Israel Ltd., an affiliate of Merck KGaA, Rehovot, Israel) according to the manufacturer’s protocol. Reverse transcription and DNase treatment were performed with the iScript™ gDNA Clear cDNA Synthesis Kit #172-5035 (Bio-Rad Laboratories Ltd., Rishon Le Zion, Israel). In order to detect genomic DNA contamination, the cDNA was amplified using ACTIN primers 5′-TTGCTGACCGTATGAGCAAG-3′ (Forward) and 5′-GGACAATGGATGGACCAGAC-3′ (Reverse), which differentiate between genomic DNA and cDNA sequences. The cDNA was amplified using the ProFlex PCR System (Applied Biosystems by Thermo Fisher Scientific, Airport City, Israel). To investigate the effect of the mutations located in exon junctions in the genes *SlCcd7* and *SlDWARF14* on the transcript, the cDNA of these genes was amplified and sequenced using the primers 5′-TCTTAACGTTCGGGTCGTCG-3′ (Forward) and 5′-GGTACAGAGTGGTCCCTTGC-3′ (Reverse) for *slDWARF14*, as well as 5′-TGAATGGAACAAAGCAGCAG-3′ (Forward) and 5′-GTTGGTAGGAGCCCAAAAGC-3′ for *SlCcd7*.

Quantitative polymerase chain reactions were performed using the Applied Biosystems™ Fast SYBR™ Green Master Mix on a StepOnePlus™ Real-Time PCR System (Applied Biosystems by Thermo Fisher Scientific, Airport City, Israel). Cycling conditions were 95 °C for 20 s, followed by 40 cycles of 95 °C for 3 s and 60 °C for 30 s and fluorescence acquisition at 60 °C. The relative mRNA level was determined for each gene in three biological replicates. The gene ACTIN (using the primers described above) served as a control for normalization. *slD27* was amplified using the primers 5′-TCCCTAAGCCTATTCTTTCTCTG-3′ (Forward) and 5′-TCACCTCACAAGGTCCAACTA-3′ (reverse); *slCcd8* was amplified using the primers 5′-CCAATTGCCTGTAATAGTTCC-3′ (Forward) and 5′-GCCTTCAACGACGAGTTCTC-3′ (Reverse).

### 4.4. Strigolactone Quantification Bioassay

The amount of SL in the mutants’ roots was estimated using a bioassay based on the germination of *P. aegyptiaca* seeds [72,73]. Three-week-old seedlings of wild type (M82) and the mutants *sb1*, *sb2*, and *sb3* were transferred from soil to a hydroponic growth system on Hoagland medium in the greenhouse for 3–4 weeks. Then, the medium was changed to Hoagland, which lacked the Pi required to induce SL synthesis. After one week, root samples (5–10 g fresh weight) were ground in a mortar and pestle with liquid nitrogen and then extracted with ethyl acetate in a glass tube. The tubes were vortexed for 10 min and then centrifuged at 12,000× *g* for 5 min, and the organic phase was transferred to a glass vial. The extraction of the root pellet was repeated once more, and the extract was dried under nitrogen gas. The dried SL samples were dissolved in sterile distilled water to adjust the concentrations equivalent to 1 g root fresh weight per 1 mL of water. The vials were stored at −20 °C. *Phelipanche aegyptiaca* (Pers.) seeds were collected from fields near Kfar Yehoshua and Mevo Hama, Israel. Dry seeds of *P. aegyptiaca* were surface-sterilized in 70% ethanol for 2 min, rinsed three times with distilled sterile water, then sterilized in 3% NaOCl for 3 min, and again rinsed three times with sterile distilled water. Seeds of *P. aegyptiaca* were placed for three days in six-well Elisa plates (Nunc, Roskilde, Denmark), moistened with 0.6 mL of sterile distilled water on a fiberglass filter paper (GF/A Whatman, Maidstone, UK) at 25 °C in the dark. After three days, the water was removed, and 200 µL of root extracts was applied to the disks. This amount was determined after the calibration of the bioassay with root extracts from M82 roots that were applied in different dilutions (Figure S7). The treated seeds were incubated in the dark at 25 °C for two weeks. All the germination treatments were conducted under aseptic conditions. The germination response of the *P. aegyptiaca* seeds was observed 14 days after stimulation by the root extracts. Dry seeds moistened only with deionized water were used as a control. Water alone did not induce any germination. Germinated and non-germinated seeds were counted under a microscope.

## 5. Conclusions

In the last two decades, strigolactones (SLs) have emerged as key signaling molecules in plant development and interaction with symbiotic soil fungi. These phytohormones, primarily synthesized in the roots, play a pivotal role in regulating plant architecture in response to nutrient availability in the soil by reducing shoot branching and enhancing lateral root growth.

Our quantitative data on isogenic tomato mutations that impair SL biosynthesis and signaling yielded substantial conclusions on SL function in an important crop plant. The yield of grafted tomato plants unveiled a significant SL biosynthesis in the shoots that revokes the excessive branching phenotype resulting from SL deficiency in roots. This phenomenon was proven valuable by utilizing a SL-deficient rootstock resistant to the broomrape *Phelipanche aegyptiaca*.

Our study uncovered a feedback process controlling the steady-state level of SL in roots that links the SL signaling pathway. This mechanism showcases the plant’s ability to fine-tune its SL production in response to internal cues, ensuring optimal growth and development.

Lastly, through grafting experiments, we have shown that elite tomato varieties grafted onto SL-deficient rootstock exhibit resistance to infection by the parasitic plant *P. aegyptiaca* without a yield loss, demonstrating a practical application for mitigating the global threat of broomrape infestations, as this method offers a sustainable approach to safeguarding tomato cultivation.

## Figures and Tables

**Table 1:** Mutations identified in the over-branching tomato mutants.

Gene	Locus	Mutation	Impact of the Mutation
Carotenoid cleavage dioxygenase 7 (*SlCcd7*) Solyc01g090660	*sb1*	G→C Position 3268 G→T position 3272	Intron/exon #7 junction, deletion of 7bp in the mRNA of *Ccd7* exon #7
Carotenoid cleavage dioxygenase 8 (*SlCcd8*) Solyc08g066650	*sb2*	G→A Position 2659	A change from Glu529 to Lys
α/β-hydrolase receptor Dwarf14 (D14) Solyc04g077860	*sb3*	G→A position 1582	Intron/exon #2 junction, deletion of 17 bp in the mRNA of *DWARF14* exon #2

## Data Availability

Data are contained within the article and Supplementary Materials.

## Supplementary Materials

The following supporting information can be downloaded at: https://www.mdpi.com/article/10.3390/plants13111554/s1. Table S1. Characterizing morphological traits in grafted plants with reciprocal combinations of the mutants *sb1* and *sb3* and M82 (Scion/rootstock). Data represent an average of 15 independent replications (±SE), conducted in the west region of Akko research station, 2018. Figure S1. (A) The branching pattern of the wild type (WT) M82 line and its isogenic mutants *sb2* and *sb3*. The branching phenotype of *sb1* is identical to *sb2*. (B) Typical plants of M82 and the mutant *sb1* at harvest stage. Figure S2. Infection of the tomato by P. aegyptiaca. Plants of sb3 (left bottom) next to WT grown in a field contaminated with P. aegyptiaca, two days before harvested. Figure S3 (A) The genomic sequence of the gene *slCcd7* (Solyc01g090660) and mutations in *sb1*. Exons are highlighted in yellow. The two mutations in the mutant *sb1* are indicated. An alternative splicing with a cryptic splice site creates a seven-nucleotide deletion in the mRNA (underlined in position 3269–3275). (B) The amino acid sequence of the CCD7 polypeptide in tomato. Figure S4 (A) The genomic sequence of the gene *SlCcd8* (Solyc08g066650). Exons are highlighted in yellow. The G to A mutation at position 2659 in *slCcd8* from the mutant *sb2* is indicated. (B) The amino acid sequence of CCD8 and the glutamate to lysine mutation in *sb2*. Figure S5. (A) The genomic sequence of the gene *slDwarf14* (*slD14*) (Solyc04g077860). Exons are highlighted in yellow. The G to A mutation at position 2582 in *slD14* from the mutant *sb3* is indicated. The 17 nucleotides sequence deletion in the mRNA, resulting from an alternative splicing is underlined. (B) The amino acid sequence DWARF14 in wild type and sb3. Figure S6. Growth habit of WT (M82), sb1 and sb3 mutants in different grafting combinations (scion/rootstock). Figure S7: Calibration of SLs measurement based on *P. aegyptiaca* seed germination induced by root extracts from M82 (wild type) in differential dilutions. The dilutions were applied with water, and the control represents water without the root extract. Germination was recorded after 7 and 14 days.
