# Supplementary material for: Tomato Mutants Reveal Root and Shoot Strigolactone Involvement in Branching and Broomrape Resistance
Source: Plants (Basel). 2024 Jun 4;13(11):1554. doi: 10.3390/plants13111554 (PMC11174905; doi:10.3390/plants13111554)

Supplementary materials

“Tomato mutants reveal root and shoot strigolactones involvement in branching and broomrape resistance.”

Uri Karniel, Amit Koch, Nurit Bar Nun, Dani Zamir2 and Joseph Hirschberg

Table S1. Characterizing morphological traits in grafted plants with reciprocal combinations of the mutants *sb1* and *sb3* and M82 (Scion/rootstock). Data represent an average of 15 independent replications (±SE), conducted in the west region of Akko research station, 2018.

Trait	M82/M82	M82/*sb1*	*sb1*/M82	*sb1*/*sb1*	M82/*sb3*	*sb3*/M82	*sb3*/ *sb3*	*Sb1*/ *sb3*	*sb3*/ *sb1*
Plant Weight (kg)	0.53±0.028^{a}	0.43±0.025^{a}	0.51±0.02^{a}	0.45±0.02^{a}	0.42±0.025^{a}	0.48±0.025^{a}	0.29±0.025^{a}	0.43±0.026	0.41±0.026^{a}
Stem Length (cm)	38.5±2.01^{a}	36.6±1.8^{ab}	33.53±1.93^{abcd}	30.15±.1.93^{abcd}	35.5±1.8^{abc}	26.4±1.8^{d}	25.4±1.8^{d}	28.7±1.86^{bcd}	28.03±1.86^{cd}
Branching/Stem length ratio	1.7±0.33^{d}	1.78±0.29^{d}	1.95±0.31^{cd}	3.98±0.31^{a}	1.91±0.29 ^{d}	3.30±0.29^{abc}	3.33±0.29^{ab}	2.46±0.3^{bcd}	3.39±0.3^{ab}
Fruit yield/plant (kg)	10.04±0.083^{a}	8.54±0.72^{a}	7.72±0.72^{ab}	4.22±0.79^{cd}	6.94±0.75^{abc}	4.3±0.83^{bcd}	3.66±0.88^{cd}	3.84±0.79^{cd}	3.58±0.72^{d}

Figure S1: A. The branching pattern of the wild type (WT) M82 line and its isogenic mutants *sb2* and *sb3*. The branching phenotype of *sb1* is identical to *sb2*. **B.** Typical plants of M82 and the mutant *sb1* at harvest stage.

A

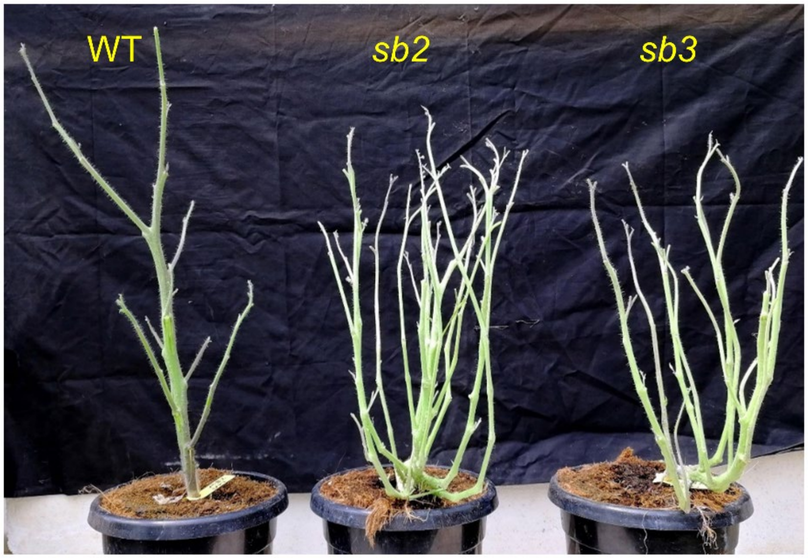

B

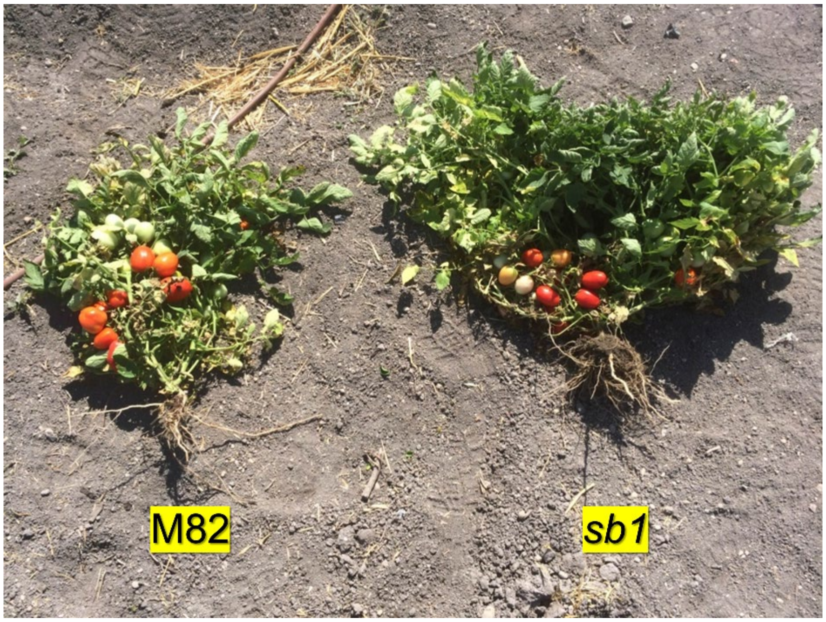

Figure S2. Infection of the tomato by *P. aegyptiaca.* Plants of *sb3* (left bottom) next to WT grown in a field contaminated with *P. aegyptiaca*, two days before harvested.

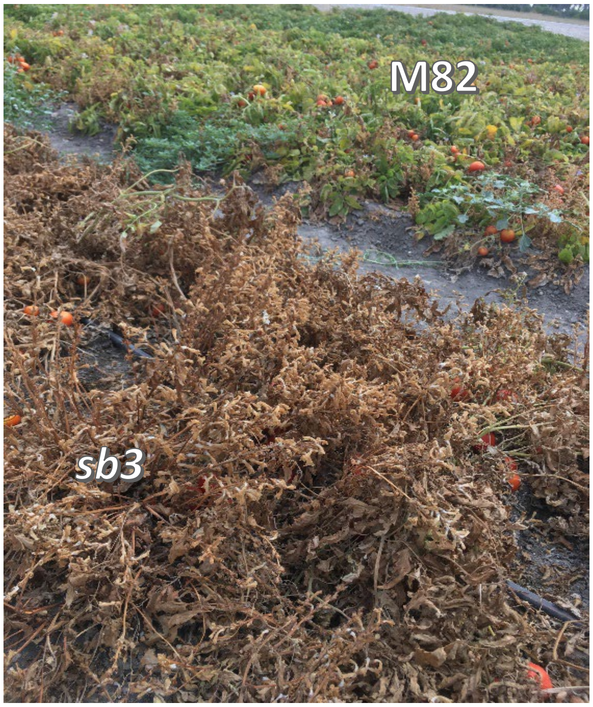

Figure S3 A: The genomic sequence of the gene *slCcd7* (Solyc01g090660) and mutations in *sb1*. Exons are highlighted in yellow. The two mutations in the mutant *sb1* are indicated. An alternative splicing with a cryptic splice site creates a seven-nucleotide deletion in the mRNA (underlined in position 3269-3275)

ATGGATCTTCAATTTGTATCACTACCACCCAACTCTAAAACAAAAGCCAAAATGCAGGCCAAAGCTTGCCATAATAT
TAACAATATTCCTCCAAAACTCTTGCCACCGGCTAAACTGCCGTCCACGGTGGCGATGAGCCCGAGCCAATTAACAT
TGCCTAGCCACGTGGCGCGAGCCATAACGATCACCACGTCCCCAACTCATGAAGTTTACACACCAGAAATCGATGAC
ACAGTTACTGCCTATTGGGATTACCAATTCCTTTTCGTGTCCCAACGTTCTGAAGCTACCGAACCCGTTTCACTTCG
GGTCGTGGAAGGATCCATACCATCCGATTTCCCTTCCGGTACGTATTACCTCACCGGGCCGGGCCTATTCGCGGATG
ATCATGGTTCCACGGTGCACCCTCTAGACGGACACGGCTACTTAAGGACATTTGAAATTGATGGTAGTACGGGTCAG
GTTAAGTTTATGGCTAGGTACATTGAAACGGAGGCTCAGACTGAGGAGCGGGACCCGGTGAGTGGAAAGTGGAGGTT
CACTCACCGGGGCCCGTTTTCGGTACTGAAAGGAGGGAAGATGGTTGGTAATACGAAAGTTATGAAGAATGTGGCGA
ATACTAGTGTGTTACAATGGGGTGGTAGGTTGTTTTGTTTGTGGGAAGGTGGTGATCCTTATGAAATTGATTCTAAA
ACTTTGAATACACTTGGAAAATTTGAATTAATTAAGAACTCTGATCAAGTATTAGAAGATAAAAAAATTAGTCATAG
TGATTTTTTGGATGTTGCTGCTCAGCTATTGAAGCCTATATTATATGGTACGATACGAAAGGACTTTTATTGTGCTA
AATAATGAAAATTGATCTTTATTTAATGACGAATTAGCTAAATATTTTTATTTGCAGGGGTGTTTAAAATGTCTCCA
AAGAGATTGTTATCTCATTACAAGATTGATACTCGTAGAAACAGACTTTTAATCATGTCATGCAACGCAGAGGATAT
GTTGCTCCCTAGGAGTAATTTTACATTTTATGGTAAGATTGTTTAATTTTATGAACTGAAAATTCCATTTAATTAAT
TTCTTATGTTTCTTCCTTTTTAATGTTTAAAGTATCTATTGCACTAGCATTCATGTCTGGTGTAGTGTAAATTAGGT
CATTATTTTAAATAAAGAAATCGATTTTATAAAGTTCATATTAAAGAAACGATTCATTCCTTAAAATCATCGATGTA
AAATTTTTCTTATCAGTAAATTAAAGCAACATCAAAAATGAAAGGAAAAACAAGCTTTTTCATATTATTTTGGCTTT
TTTTTTTTGCTTGTAAGCCTGACATCAGTAAGGTGGGATTTTATTATATGTTTTCCTTTTTTTGAAAATAAATAAAT
AAATAAGAGTAGAGGGTCCAGTGATAAATAAAATATTTATAATGTTTTTAAACTAAAACAAGGAGTAAACATAATTA
AATGATCATTATCCTAGTATATAATACTCCAATAATCCAACTCTACTTTTTAAAAAATTGTATATTTTCATCAACAT
CACATGTAGATGTAGTACAACGTTAACAAGAATAATTCTTTTGTTTTTGTCTCCATATATTCATAATAATTCAAATT
CTAGTTAATTTAATTCATTTTATTTTTTTTTTGTCTAGAATTTGATTCCAATTTCCAGCTACTACAAAGCCAAGAAT
TCGAGATCCCAGATCATTTAATGATACATGATTGGGCTTTTACTGATACTCACTATATATTGTTCGGCAACCGCATC
AAACTCGATATCCCCGGTAACTAATTATTCGATTTCTATACTAAAAATAAATATTTCATTTTACTTATTTATTCACT
AAAATTTTGCGTACGCCATCGTTGCAGGATCAATGACAGCAGTATGTGGGCTTTCTCCAATGATATCAGCATTATCA
GTAAATCCAAGCAAACCAACATCTCCAATTTATTTGCTGCCTAGATTTCGTAACAATAATGTAGAAAGAGATTGGAG
AAAACCTATAGAAGCTCCTTCACAAATGTGGGTGTTACATGTTGGAAATGCTTTTGAAGAAATTGATGAACAAAATG
GAAATCTAAACATACAAATTCAGGCTTCTGGTTGCTCTTACCAATGGTTCAATTTCCAAAAAATGTTTGGTAAGCAA
GCACGAGTTTATGTCTGCCGCACTGTCAGTATATATAAACTAAAACGTTTTATATAGTAGATGATTAAAACTTAATT
TGTTTCTTTTTCTCCATATATATTTTTCAGGCTATGATTGGCAAAGTGGTAAACTTGATCCTTCCATGATGAATGTA
GAAGAAGGAGAAGAAAAGCTATTGCCTCACTTAGTTCAGGTCTTCTTCCTAATGTTTAGTTTATTATAATAAAATCG
TAAAATTATTATTTTTAAAACAAAAAATAAAATAAATAAAAGACGATTGGACTTAATCAATACATATAATCATTTTA
TTTCATGTGACACATATGATGATACATGAGAAACTTTTTATTTTTTTAAAGATGTTTCAGAATCGGGTCGTTATTAT
TATTTTATAGTAGTGTCACGTGTAATGCGAATTTGTGATCATGGGTATGCAGGTATGTATAAACTTAGATAAAAAAG
GGAATTGCACAAAATGTTCAGTAAATGATCTAAATCCTGAATGGAACAAAGCAGCAGATTTTCCAGCGATGAATCCA
GAATTTTCAGGCAGAAAAAACAGATATATTTATGCAGCAACATGTACAGGTTCACGTCAAGCGCTACCCCATTTTCC
TTTTGACGCAGTCGTGAAATTAAACGCGGTTGATAAATCAGTCCAAAAGTGGTCAGCTGGTAGAAGAAGATTCATTG
GTGAACCTGTTTTTATTCCAAGAGGAACTAATAAAGAGGATGATGGATACCTTCTTGTGGTTGAAGTAAGTAATTAT
AACTTACATATACACATACACTAATAAAGTTTGCATAGTTAGTGTCTTTTAGCACCACATGTCTTATTTTTCTACGA
GTAAAATATCACTTTTAGTTACTTTGACTTTAAATATGTTATTTTCGAGTGATATATGATTATGTAAGAAAATTTTA
GAAGTTTACAATGTGGTAAATTTTTATACTCTAGTGTCACCTTTATTTGTTATAGCAGACAATTCGTCTTAATTCAA
AGATTGACAAATTAAACGTCGTATTTAAAAGACATTAATTGTAATTTATATATAAAAAAAATAGTATTACTTCTATT

sb1 **C** **T**

CTTATGATTTTGTGTGTGAATATTTAATTTGCA**GTATGCAG**TGTCAACACAAAGGTGTTATCTTGTAATTTTGGATG
CACAAAAAATTGGAGAGAAGAATGAAGTAGTTGCAAGACTTGAAGTCCCAAGACATTTGAATTTTCCTCTTGGTTTT
CATGGCTTTTGGGCTCCTACCAACTCTAGCCTAGCCAATCTACAAAAAATTGAATCCAAGTGTAAAAACTCTTGGTC
AATGATGAAGGATAACATGGTTAAACTTGGACAATAATAAAAAAAATAATAAAATAAATAATGTTTTTATATATATA
GATGTTCATTAATAGCTTAATTATGCAAAAGTTTTTTAGAAAATTTAATAAGGTTAATTTTTTGGTAAAATAGTTCA

TTTGTATACTAGAATCCAGAAGAGTTCCAACAATGTCAATTCTTTTATTTCAGATGTAAGCATTTTTTTAGTTGTAT
AAGCTATGATCATATAATTAATTTATTTTG

Fig. S3 B. The amino acid sequence of the CCD7 polypeptide in tomato.

Wilde type

MDLQFVSLPPNSKTKAKMQAKACHNINNIPPKLLPPAKLPSTVAMSPSQLTLPSHVARAITITTSPTHEV
YTPEIDDTVTAYWDYQFLFVSQRSEATEPVSLRVVEGSIPSDFPSGTYYLTGPGLFADDHGSTVHPLDGH
GYLRTFEIDGSTGQVKFMARYIETEAQTEERDPVSGKWRFTHRGPFSVLKGGKMVGNTKVMKNVANTSVL
QWGGRLFCLWEGGDPYEIDSKTLNTLGKFELIKNSDQVLEDKKISHSDFLDVAAQLLKPILYGVFKMSPK
RLLSHYKIDTRRNRLLIMSCNAEDMLLPRSNFTFYEFDSNFQLLQSQEFEIPDHLMIHDWAFTDTHYILF
GNRIKLDIPGSMTAVCGLSPMISALSVNPSKPTSPIYLLPRFRNNNVERDWRKPIEAPSQMWVLHVGNAF
EEIDEQNGNLNIQIQASGCSYQWFNFQKMFGYDWQSGKLDPSMMNVEEGEEKLLPHLVQVCINLDKKGNC
TKCSVNDLNPEWNKAADFPAMNPEFSGRKNRYIYAATCTGSRQALPHFPFDAVVKLNAVDKSVQKWSAGR
RRFIGEPVFIPRGTNKEDDGYLLVVEYAVSTQRCYLVILDAQKIGEKNEVVARLEVPRHLNFPLGFHGFW
APTNSSLANLQKIESKCKNSWSMMKDNMVKLGQ

Mutant sb1

MDLQFVSLPPNSKTKAKMQAKACHNINNIPPKLLPPAKLPSTVAMSPSQLTLPSHVARAITITTSPTHEV
YTPEIDDTVTAYWDYQFLFVSQRSEATEPVSLRVVEGSIPSDFPSGTYYLTGPGLFADDHGSTVHPLDGH
GYLRTFEIDGSTGQVKFMARYIETEAQTEERDPVSGKWRFTHRGPFSVLKGGKMVGNTKVMKNVANTSVL
QWGGRLFCLWEGGDPYEIDSKTLNTLGKFELIKNSDQVLEDKKISHSDFLDVAAQLLKPILYGVFKMSPK
RLLSHYKIDTRRNRLLIMSCNAEDMLLPRSNFTFYEFDSNFQLLQSQEFEIPDHLMIHDWAFTDTHYILF
GNRIKLDIPGSMTAVCGLSPMISALSVNPSKPTSPIYLLPRFRNNNVERDWRKPIEAPSQMWVLHVGNAF
EEIDEQNGNLNIQIQASGCSYQWFNFQKMFGYDWQSGKLDPSMMNVEEGEEKLLPHLVQVCINLDKKGNC
TKCSVNDLNPEWNKAADFPAMNPEFSGRKNRYIYAATCTGSRQALPHFPFDAVVKLNAVDKSVQKWSAGR
RRFIGEPVFIPRGTNKEDDGYLLVVE**CQHKGVIL***

Figure S4 A: The genomic sequence of the gene *SlCcd8* (Solyc08g066650). Exons are highlighted in yellow. The G to A mutation at position 2,659 in *slCcd8* from the mutant *sb2* is indicated.

TCACCATACTCTCAAATTCTCTCAAATAATATTCTTCACATCTATGGCTTCTCTTGCTTCTTCAACAACCAAAATTT
ATTGTAACAAGATCCTTCCTGACATGTTTGATCATGGCAAACATGAATCTCATCTTGGATCAAAGTTGAAAAACAAC
GAAAAAAACAAGAAAAAAATTGGACTTGAAATTGGTTACAAAGGTTGCTAGCCAATTGCCTGTAATAGTTCCACCACC
AGATCAAGAGGTGATTAGTAAGGAGAAAAAGCTTGCTGCATGGACTAGCGTACGCCAAGAAAGATGGGAAGGAGAAC
TCGTCGTTGAAGGCGAGTTACCATTGTGGCTGGTATGTTATTGTTTGTGTATATAAGTTAAATATGAACGTTTCGTG
TTACATCCATTTTTTTGTTTTAATGTGAAGTTTTTCTTTTTTTGGAATCACTTATATATTATGATTTTTCTTCACCAA
AAAGTATTGAGATCAAAGATGCATGTGTACCTAATACATGTTTTTCTCTCAAGAAAATATGAAGTATACATATTGAA
AACAAAGGTTTATGAACTTAATTATTCTGCTATTTAGTACACTACTATAGACCCAAAGATTTTCCTCACTTGATAAT
CAGTCGGAAATTTTGTATTATAAAATATGATTTTCTGAGTCATTTTCGGCCGAGTTAGCTTACTGAAAAAAATGTGT
GACGGAAAAGAGATTCTCACTAAAATATATAGTAGAAAAGTTCTAATATTTCTCCATGAAAACATTACTACTTTTTC
TTTCTTATCAATTCAATTAGAAAAATCGACGAAAATAGTCGCTAAACATTTTCAAATAAGAAAATAATGATTTCTTT
AATAGTGGGTATAAACACTCGTCAAGAAGCTAATTAATAAGAAGCAATTATATTCTTAGCAATAATCTTGTGATGAT
CTCACATTCAACAAAGGATATTCTCTTCTTTCAAGATGATAAATAATGTATAGTATTAAAACGACAAGAACTATTTA
AGATCTGATTGAAGATTAAAAATAAATAAAACAATCTTTTTCCTTCGTATATAAAATATTTCTTTTGGAAAAATAAA
ATATTCCTTTATCTTTTGTCTGAATAAATTTAATAGGACGTAGTACATCAGCTTAACCTCATTCCACATCATGTCAC
ATTCATTCCAAGTCCAAATTTGAGCACATAAAATTATTTTATTTCATTATTTTCTTTGTCTATGTAGAATGGCACGT
ACCTAAGAAATGGTCCAGGACAATGGCACATAGGTGACTACAATTTTCGTCACCTTTTCGATGGCTACGCTACCTTA
GTCCGTCTTCATTTCGAAAATGGACGATTAATCATGGGTCATAGACAAATCGAATCGGACGCATATAAAGCAGCAAA
AATCAGTAAGAAAATATGTTACAGAGAATTTTCAGAAGTACCTAAAGTAGACAATTTCTTATCCTACATAGGTGACA
TGGCAAAATTACTCTCCGGTGCATCCCTAACCGATAATGCTAACACTGGAGTCGTTAAACTTGGGGATGGACGCGTA
GTCTGCTTAACTGAGACGATAAAAGGTTCCATTGTAATTGATCCGAACACCCTAGATACAATTGGGAAATTTGAATA
TAGTGACTCGTTAGGTGGATTGATTCATTCAGCTCATCCAGTGGTTACGGACAGTGAGTTCATAACGTTGATTCCGG
ATTTAATGAACCCGGGATATACGGTGGTGAGAATGGAGGCAGGGACAAATGAGAGGAAGTATATAGGGAGAGTGAGT
TGTAGAGGAGGACCAGCACCAGGATGGGTTCATTCATTTCCTGTTACAGAAAATTATGTTATTGTGCCTGAGATGTC
ACTAAGGTATTGTGCAAAAAATTTGTTGAAGGCTGAGCCAACACCACTGTATAAGTTTGAGTGGCATCCTGATTCTA
AAGCATTTGTACATGTTATGTGTAAAGCCAGTGGCAACATTGTAAGTCATTCCTTTCTCATCACTTAATTCTTTTAT
GATGTAAGAGTAATCTAATGGTGAGAGACTATCTATTCAAAAGGTGGCAAGTGTAGAAGTGCCATTATACGTGACAT
TCCACTTCATCAATGGATACGAAGAAAAAGACGAAGATGGAAGAGTTACCGCTGTGATTGCAGATTGCTGTGAGCAT
AGCGCAGACACCACCATCCTTGACAAGCTCCGCCTTGAGAATCTTCGTTCCTTCAACGGCAAGGATGTCTTACCTGA
TGCAAGGTACAATTGAAGGAAATGAGTATTACATCACTTCAAAAGTACATTATTAATGGTGTTTGGTGATATATAGG
GTTGGAAGATTCAGAATACCATTAGATGGAAGTCCATATGGAGAATTAGAAGCAGCATTGGATCCAAATGAACATGG
AAAAGGCATGGATATGTGCAGTATGAATCCTGCTTATTTAGGCAAGAAATACAGATATGCTTATGCTTGTGGTGCTA
AGAGGCCTTGTAATTTCCCCAACACCCTCACCAAGGTCAGCTTCTTTTCCTTACAATGGTTATTGTTTCATCATCAA
GTAATAACTGACAATATTTTGCAGATTGATTTATTTGATAAGAAGGCAAAGAATTGGTATGATGAAGGTGCTGTGCC
sb2 **A**
TTCTGAACCATTCTTTGTGGCTCGACCCGGTGCAACAGAG**G**AAGATGATGGTAATTAGTTTACCAAAGGTCATTTGG
CCCTGTTCTGAACAAGGTGTAACATTTTTGAACTATATATGCAGGTGTTGTAATCTCAATGATCAGTGACAAGAATG
GAGAAGGATATGCTCTAATACTGGATGGATCAACATTTGAAGAAATTGCAAGAGCTAAATTTCCTTATGGTCTCCCC
TATGGGCTACATGGTTGTTGGGTTCCAAAGATATAGTATCACCCCACATCCAAGACAATGTTATATCATATTAGCCT
AATACAATCTACAAGTTATAATAAGATGATATTACTAAATTTTCCAGTTTTTGAAATAGCTATTAGTGTACTATTTT
GTGTATATTTGCTCTCAGTAATAGAATGTGTTAAACCATATTAATAGTATGAATGCATATACTCTAAAATGA

Figure S4 B: The amino acid sequence of CCD8 and the glutamate to lysine mutation in *sb2*

MASLASSTTKIYCNKILPDMFDHGKHESHLGSKLKNNEKNKKKLDLKLVTKVASQLPVIV 60
PPPDQEVISKEKKLAAWTSVRQERWEGELVVEGELPLWLNGTYLRNGPGQWHIGDYNFRH 120
LFDGYATLVRLHFENGRLIMGHRQIESDAYKAAKISKKICYREFSEVPKVDNFLSYIGDM 180
AKLLSGASLTDNANTGVVKLGDGRVVCLTETIKGSIVIDPNTLDTIGKFEYSDSLGGLIH 240
SAHPVVTDSEFITLIPDLMNPGYTVVRMEAGTNERKYIGRVSCRGGPAPGWVHSFPVTEN 300
YVIVPEMSLRYCAKNLLKAEPTPLYKFEWHPDSKAFVHVMCKASGNIVASVEVPLYVTFH 360
FINGYEEKDEDGRVTAVIADCCEHSADTTILDKLRLENLRSFNGKDVLPDARVGRFRIPL 420
DGSPYGELEAALDPNEHGKGMDMCSMNPAYLGKKYRYAYACGAKRPCNFPNTLTKIDLFD 480
sb2 **K**
KKAKNWYDEGAVPSEPFFVARPGATE**E**DDGVVISMISDKNGEGYALILDGSTFEEIARAK 540
FPYGLPYGLHGCWVPKI 557

Figure S5 A: The genomic sequence of the gene *slDwarf14* (*slD14*) (Solyc04g077860). Exons are highlighted in yellow. The G to A mutation at position 2,582 in *slD14* from the mutant *sb3* is indicated. The 17 nucleotides sequence deletion in the mRNA, resulting from an alternative splicing is underlined.

TACTAATAAAAATGATTAATGATGGTGACTGGTGAATAATAATTTTGATAAAATAATACTGAAAATTATTTATACAA
AGTATTAACTCTTAATTAATGCTATTAAAACTTTATTGAAGATATAAATGATTTGGATATATTTAAATTTAATAAAA
TTTTAATCTACTTAAACAATTTTATCTTAACAGTCTAATATTATACCATTCAAAATTTAGAACTCCTTAAAAAATGG
GGGCTTATACTCCATTACAATCAAACATAAGTGGCCATGGTTATATTTAACGGAAAAATACAATTTGTATAGATTTT
TTTTTAAAAAAACATACAAATTGGAGAAAGAATGAATAGTCAATGGTGTTTTTTTTTCCGATTTAAACGTTTTACGT
GTTTTTCACAAGTAAAGTATAATTACTATCATATAATATTACGTACGTACAAAAACTAATAGTACCAAATTGATTAT
TTCTTGTTGATAATTAAAGAACCAAAAACCCTTCAATTAAAAAACACACTCACTTTTCTTTTTAGTTAATCTTAAAA
TACATTTCTATATTTCAAAATAATTTAGCTTTGAAATGTCTATTTTTTTTTCTTAGTGAGATGATGTATCGTCATAT
TTTAGACCATAAATTTTACTCCCTGCGTTTCTTTTTAGTTGTAATAGTTTCATTTTTTAGATTCCAACAATATAAAT
TTTGACTAACATTTTAAAATATATACTTTCATTAAATTGATATGAAAAAAACAATTTATAGTAATTTCTGTATAATT
TTTCAATATCTAAAATTTTATTTTAAAAATATAATTGAAAGATTTGAAAACAATCAAATTAACTCTAAAAAATATAA
TATAACAATTAAAAGGAACAAAAATAATAAAAAAGGTCTTTTCTAAATTCTATATATAAAAAAAATAGCTTATATAA
ATTAAGACGGATGAAAAGCAAGTAGGTGTCTTTTGGCAACTTTATAAGTACCACACCCCTCCAATCACTTTTTCCCA
TTCACACAACCATTAAAAAAAAAAAAAATTCAAAAAAAAAAGAAAAAGGATTTAAGTATTCTTCATTTTTTTTCGTCC
AATAAAAAAAGGAACCAAAAAACTGAAA**ATG**GGTCAGACCCTTTTAGATGCTCTTAACGTTCGGGTCGTCGGTTCCG
GCGAAAGAGTTTTGGTTTTAGCCCATGGGGTCGGTACCGACCAATCCGCTTGGAATCGAATTTTACCTTTTTTTCTC
CGAGATTACCGTGTTGTTCTGTACGACCTTGTCTGCGCCGGCAGTGTAAATCCTGATTTCTTCGATTTCCGACGTTA
TACGACACTTGACCCTTACGTTGATGATCTTCTACATATTCTCGATGCTCTTGCAATCGATCGTTGTTCCTATGTCG
GACACTCTGTCTCCGCCATGATCGGAATTCTCGCTTCGATTCGCCGCCCTGAACTCTTCTCTAAACTCATCCTCATC
GGAGCTTCGCCCAGGTGAATTTTCTTTTTTTAAATTTTAACTTTTTTTTTTACTTATTCCATCTCTTTTGTAATAAC
TCACAAATCATAGGAGAAATATATAAATCTCCTAGGTAAGGTGTTATTTATCCACTTTTAGTTTATTCGAGTTAATT
TAAATCAATATTAAAATATAAATATTTAAAAATGTATACTATTAATTAAATTTTATGAAATTTAATTTTTAATTAAT
AAAAAATAACACATAAATTAAAATAGTAAAAGAATCATACTATTATTTACCAATTTGTAGAAAATGGGGAATGTGGA
TTATTTTTAAAAAAAATTACATCCAATATTTGAGTCAAATTAATAAGTACATCACACATGTATTCAAATAACAAATG
AAATAAGTAAATGTATTGTGGTAAATTCTTTAAACTTTGACATTTAAATACAGCACATTTATGTACTTTAATTTTGG
AGAGTTAAAATATCATTTGAGTTTGTTTTTTTTAAAAAAAATTGATTTAATAAAAAAAAATTGTTATATGACATATA
TATGTGTATAATATCCTTCTGAACATGAATATATTGGAACCAAACTCTGTACCCCATAAAATAATAGTAAATTTTGT
TTATGTCGTACTATTTTTAAATATCGTTTGCATATATTACGAATTATGGTATTATTATTTTTTGCATATATAGTATT
TTTTGAGTAAAATTTATGAAATCGACTCTGTATTGACAAAGTAACAATAGAATCTATTTATATTGTACTTTTCGTTG
ACTTCAATTGCATTAGTAATGTTGTTCATATGTACGTATAATATTTGAGTTTCTATACATACAATATAATATTGAGT
TTGGTTGGTCTACTCAAAAATAGGAATATTTAACGTGGTCCTTTTGTTTATTTTATTTTCTTTTACTTTTTCTCAAT
TCCCCATAAAGTTGAGAGCACTTTTGTCAAACTTACTTATCTTATCCTTAGTTTATGTTTGACAAAATTCATGAATT
TATTTGCTTAGGAATGTTATGATCAATCAATTGACATTTCATGTTATCAGAATCCTTATCTTAAAATCTTTTTAACT
CCACCAAATCTTTAATTAAATAATGTTGTACACCAATTC
Sb3 **A**
A**G**ATTCTTGAATGATGAAGACTACCATGGTGGATTTGAACTCGGAGAAATAGAGAAAGTGTTTTCAGCAATGGAGGC
AAATTATGAAGCATGGGTCAATGGTTTTGCCCCGTTAGCCGTCGGAGCCGACGTTCCGGCGGCTGTACGAGAATTCA
GTAGAACATTGTTCAATATGAGACCAGACATAACATTGTTTGTGTCAAGGACAGTATTTAATAGTGACATGAGGGGT
GTTCTAGGTCTTGTGAAAGTACCATGTCATATTTTTCAGACAGCAAGGGACCACTCTGTACCCGCTTCAGTCGCGAC
GTATCTAAAGAACAACCTTGGTGGGTGGAACACCGTGCATTGGTTGAATATTGAGGGACATTTGCCACATCTTAGCG
CCCCGAATTTATTGGCTCAAGAACTAAGGAGGGCTCTTACTCATAGGTGATCGATCCTGTTGAAGCGGGTACAGAAT
GGTTGAATTCATAAAACTTAAATCCTGAATTATCATTTGTTTTGCGTCAACCAAACCAAAAACAAAGAGACAAAAGG
ACTATGGATGGTTTGTTTAATTTTTGAACCTTTGATACACTTTGTGGATTTGTGGAAAAAGTTTGGATTTTTACCCA
ATAGGATTTTGACAACATTCAAGAATAACAATCCTTGCAAAAAAGAAACTTGAATACACAAATTTTGAGTTTATTAT
TTTTGTGATGAAAAGAGGTTTTTGTTCAATTTTCTTTTTCCAATTTACTATGTCAGAATAAAGCAAAGAATATGATT
TTTATTCTTTTCATAATTGACATTCGATCATATTATAATGTGTTTACTTCACAAATAAATTGACCCAAATTTTGTGA
ATTTAAGAGTGAAAATTTTGGTTCTAATAATTTATTATGATACGAAAAATATCCTATATAATTAATATCAATCGAAT
GAGATCGAGAATAAATTTTTGAAGGAGATGGTGAGATCAGGCAAAATTTCAAGGATGGGTCCATTATGATTATCCAT
AAAAAAAGAGGATAAGATGCACATGATAAATTTCACGTGAGAAAGTGTCCCTTCTTTGGACCATTTCCACTTTCCTC
TTTATTTTTAGGGTTGTATTATTCAAATTCACTTTTGTTTTTTTTTTAAATTTTATTTGGTATTTATGTATGTAAAG
AAGAATTTGCATCTTAAAAAGAAAACATAAAGTCAAGTGGTACAAAAATAGGAATTTTTAACCAAACTAAGTCATTA

TATAAAACAATTTACTAAAGTAATATATTTTTCTAAAATTTTACAAAACTAGTATAAACGTATTTCATGGTAACGTT
TTAGGGTATATTTTATTTTTAAAAAACTAACAGCATTAGGTTGATATACGTTACTAAAAGTAAC

Figure S5 B: The amino acid sequence DWARF14 in wild type and *sb3*

Wild type

MGQTLLDALNVRVVGSGERVLVLAHGVGTDQSAWNRILPFFLRDYRVVLYDLVCAGSVNPDFFDFRRYTTLDPYVDD
LLHILDALAIDRCSYVGHSVSAMIGILASIRRPELFSKLILIGASPRFLNDEDYHGGFELGEIEKVFSAMEANYEAW
VNGFAPLAVGADVPAAVREFSRTLFNMRPDITLFVSRTVFNSDMRGVLGLVKVPCHIFQTARDHSVPASVATYLKNN
LGGWNTVHWLNIEGHLPHLSAPNLLAQELRRALTHR*

Mutant *sb3*

MGQTLLDALNVRVVGSGERVLVLAHGVGTDQSAWNRILPFFLRDYRVVLYDLVCAGSVNPDFFDFRRYTTLDPYVDD
LLHILDALAIDRCSYVGHSVSAMIGILASIRRPELFSKLILIGASPRLP**WWI***

Figure S6: Growth habit of WT (M82), *sb1* and *sb3* mutants in different grafting combinations (scion/rootstock).

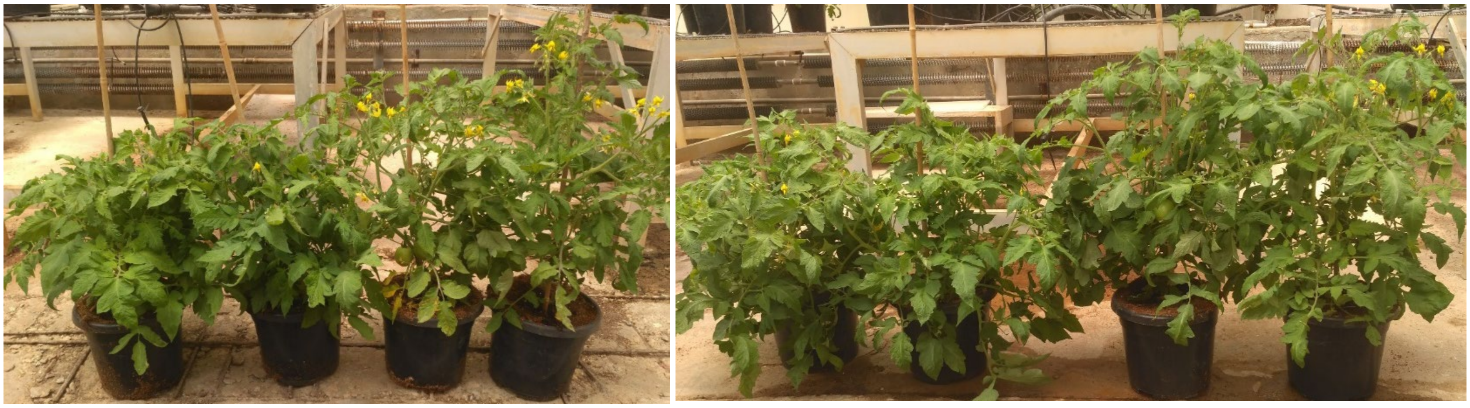

sb1/sb1 *sb1/sb3* *sb3*/M82 *sb1*/M82

Figure S7: Calibration of SLs measurement based on *P. aegyptiaca* seed germination induced by root extracts from M82 (wild type) in differential dilutions. The dilutions were applied with water, and the control represents water without the root extract. Germination was recorded after 7 and 14 days.

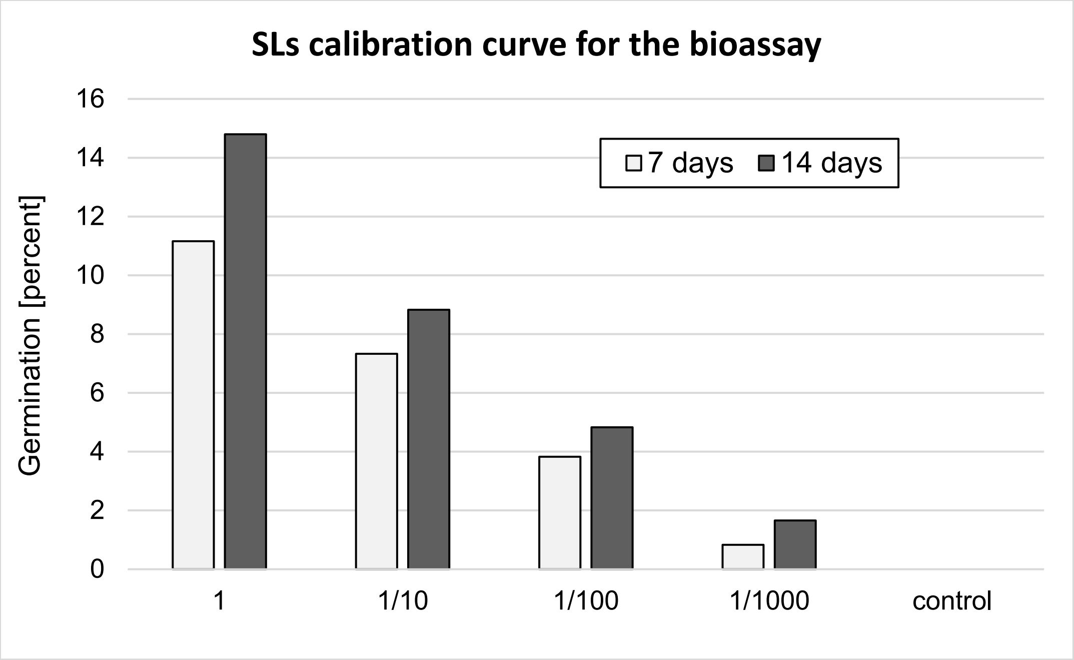

Supplement: Supplementary file 1 [file plants-13-01554-s001.zip › plants-3006128-supplementary.pdf]
